# The role of adenosinergic pathway in human autoimmune diseases

**DOI:** 10.1007/s12026-016-8870-2

**Published:** 2016-09-24

**Authors:** Ke Dong, Zhao-wei Gao, Hui-zhong Zhang

**Affiliations:** Department of Clinical Diagnosis, Tangdu Hospital, Fourth Military Medical University, Xi’an, China

**Keywords:** Adenosinergic pathway, Adenosine receptor, Immunosuppression, Autoimmune diseases

## Abstract

Autoimmune diseases are characterized by the abnormal immune response against self-tissue, which are caused by the failure of nature immune homeostasis. Nature immune homeostasis represents the normal state of appropriate immune response to nonself-antigen and unresponsiveness to self-antigens. In normal situation, immune homeostasis is regulated by immunosuppressive signal and immunostimulating signal together. Accumulating data have demonstrated that the adenosinergic pathway played key roles in immune suppression and shield body from an excessive inflammatory response. The deficiency of adenosinergic pathway results in the imbalance between the pro- and anti-inflammatory activities. Thus, researchers pay much attention to the role of adenosinergic pathway in autoimmune diseases development. To date, accumulating data have suggested an important role of adenosinergic pathway-related molecules (i.e., CD39, CD73, ADA, adenosine receptors, etc.) in many types of human autoimmune diseases. More importantly, these findings have presented potential value of adenosinergic pathway analysis to be used for autoimmune diseases diagnosis, monitoring and treatment. In this review, we will provide a comprehensive description of the role of adenosinergic pathway in human autoimmune diseases.

## Introduction

Autoimmune diseases are caused by the deficiency or losing of nature immune tolerance. Under certain circumstance, immune system attacks the self-antigens, following with excessive immune response, which leads to autoimmune diseases to happen [1, 2]. The autoimmune diseases may be restricted to certain tissues, organs or the whole body. The frequent autoimmune diseases include RA (rheumatoid arthritis), SLE (systemic lupus erythematosus), MS (multiple sclerosis), T1DM (Type I diabetes mellitus), JIA (Juvenile idiopathic arthritis), AIH (autoimmune hepatitis), etc. The basic function of human immune system is distinguishing antigens and then eliminates the nonself-antigens and protects the body from infection, tumor, etc. In normal situation, there is “alarm” signal which generates various cellular responses that aim to prevent excessive inflammatory response and restore the immune homeostasis. Several lines of evidence have demonstrated that adenosine behaves as the “alarm” signal in vivo. Adenosine activates adenosine receptors on target cells, generates multiple cellular responses and then suppresses the immune response [3, 4]. Thus, deficiency of adenosinergic pathway may result in the disorder of immune response. Recent years, researchers pay much attention to the role of adenosinergic pathway in autoimmune disease development. This present review will provide a comprehensive description of the role of adenosinergic pathway in human autoimmune diseases.

## Adenosinergic pathway regulates immune imbalance during autoimmune diseases progression

### Immune imbalance in autoimmune diseases

Autoimmune diseases arise from an abnormal immune response against self-tissues and cells and lead to the injury. In normal situation, the immune response is regulated by immunosuppressive signal and immunostimulating signal together. A fine balance between immunosuppressive and immunostimulating signals is important for maintaining the homeostasis of immune system. Accumulating data have shown that disturbed balance of immune system was involved in development of autoimmune diseases. The main T cell subsets which are pivotal for this T cell balance consist of T helper (Th) cells and regulatory T (Treg) cells, and moreover, Th cells are defined as Th1, Th2 and Th17 subtypes characterized by differential expression of certain cytokines. In SLE, increasing evidences have demonstrated that the disrupted balance between Th cells and Treg cells contributed to the disease development [5–9]. Deficiencies of Treg cells, either quantitative or functional, were found in active lupus patients, while the number of Th17 cells, as well as Th17-related cytokines, was found increased in SLE patients [10–12]. Studies have reported higher concentration of IL-17 in the serum and synovial fluid of RA patients compared to controls [13, 14]. IL-17 is the main cytokine secreted by Th17 cells, which indicated that the number and/or activity of Th17 cells was increased in RA patients, while studies showed that the percentage of circulating Treg cells in RA patients was reduced compared to healthy controls [15]. Similarly, imbalance of Th cells and Treg cells has been noted in T1DM [16]. The imbalance is manifested by expansion of Th17 cells which is concomitant with decreased number or function of Treg cells. Taken together, above data demonstrated that the disrupted homeostasis of immune system played a critical role in the autoimmune diseases development.

### Adenosine signaling pathway

**Fig. 1:**
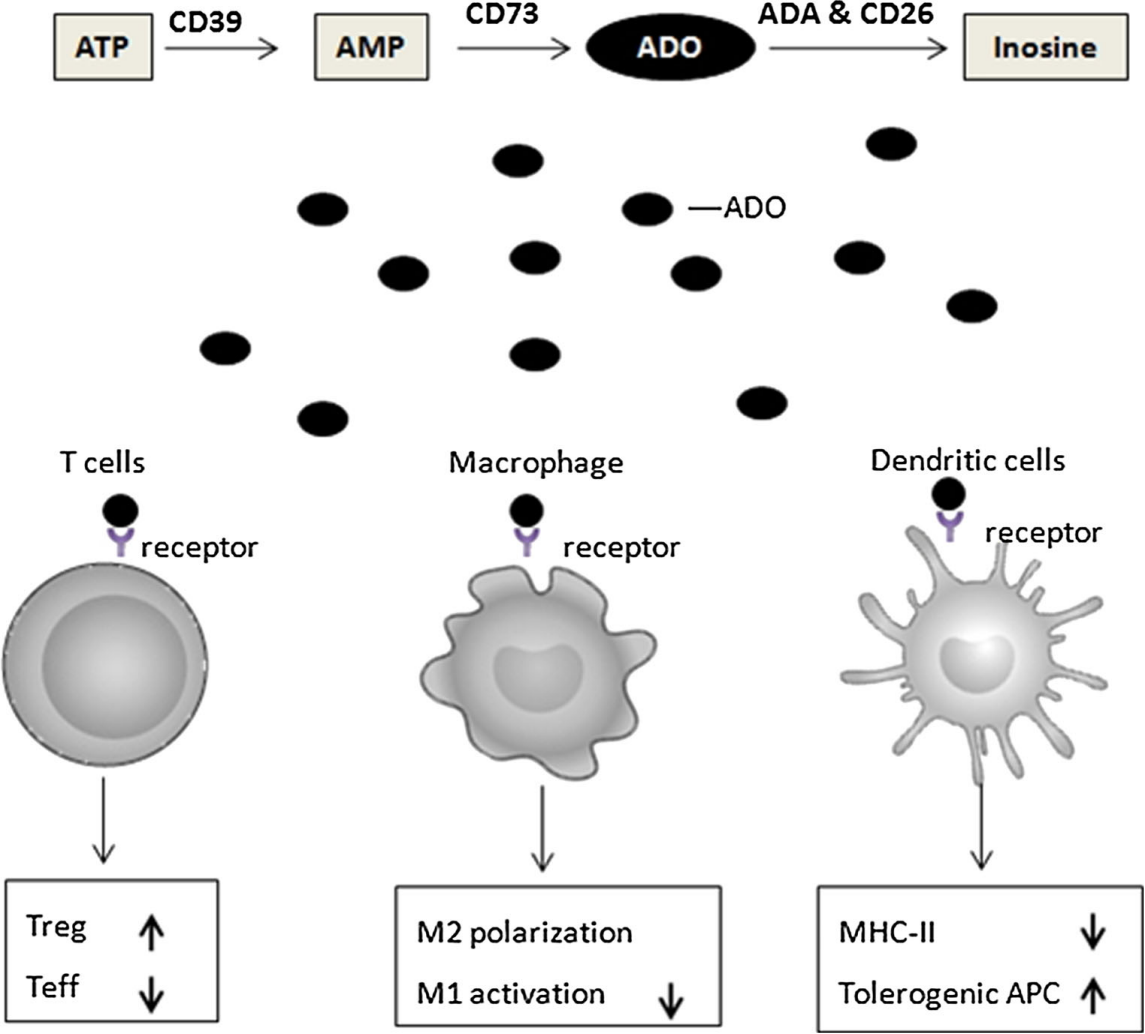
Adenosine pathway mediates immune suppression. A scheme illustrates the generation of adenosine and the adenosine receptor signaling pathway that mediates immune suppression. Adenosine is generated from degradation of ATP by CD39 & CD73. The adenosine pathway mediates the immune suppression by regulating the function of immune cells, such as T cells, dendritic cells and macrophages

Adenosine is an important immunosuppressive signal in the internal environment, which can shield cells and tissues from an excessive inflammatory response and immune-mediated damage [4, 17]. Extracellular adenosine concentration is determined by a complex ectoenzyme mechanism and uptake system [3, 18]. The generation of adenosine in the internal environment is regulated by a cascade of enzyme (Fig. 1). Adenosine is generated from degradation of ATP which is catalyzed by the enzyme cascade as follows: ATP/ADP breakdown into AMP by CD39 (ecto-nucleoside triphosphate diphosphohydrolase 1, E-NTPDase1), AMP breakdown into adenosine by CD73 (ecto-5′-nucleotidase, NT5E). Adenosine can be catalyzed into inosine by ADA (adenosine deaminase) and its cofactor CD26 (dipeptidyl peptidase 4). Uptake system, which includes ENTs (equilibrative nucleoside transporters) and CNTs (concentrative nucleoside transports), shunt extracellular adenosine into the intracellular space, thereby regulating the concentration of extracellular adenosine and terminating adenosine receptor signaling. Adenosine activates its G-protein-coupled cell-surface receptors on target cells, which generate various cellular responses that aim to restore immune homeostasis. Up to date, four adenosine receptors have been identified: A1R, A2AR, A2BR and A3R. A1R, A2AR and A2BR are conserved and highly homologous (80–95 %), whereas A3R varies substantially among different species [19]. Adenosine receptor signaling depends on the level of extracellular adenosine. Accumulating data have demonstrated that adenosine behave as an immunosuppressive signal via activating its receptors.

### Adenosine pathway mediate immune suppression

Accumulated data have shown that adenosinergic pathway-mediated immune suppression and played an important role in maintaining the homeostasis of immune system (Fig. 1). The regulatory T cells (Treg cells), also known as suppressor T cells, are a subpopulation of T cells which modulate the immune response and maintain immunological tolerance. Treg cells generally suppress immune response by suppressing the proliferation and function of effect T cells. Romio et al. showed that the suppressive function of Treg cells was dependent on the CD73 expression on Treg cells surface. They found that CD73-positive Treg cells strongly inhibited proliferation of CD4^+^CD25^−^-effective T cells in co-culture experiments, while there was no significant inhibited function when experiments were performed with Treg cells lacking CD73 [20].

Smyth et al. [21] found that Treg cells suppressed effective T cells via the production of secreted membrane vesicles, such as exosomes. Moreover, their results showed that CD73 was expressed on Treg-derived exosomes and contributed to Treg cell’s suppressive activity through the production of adenosine. The exosomes derived from CD73-positive Treg cells can significantly inhibit CD4^+^CD25^−^ effect T cells proliferation, while the exosomes derived from a CD73-negative Treg cells lack the suppressive capacity. Notably, the suppressive capacity of exosomes derived from CD73-positive Treg cell is in a dose-dependent manner. Romio et al. reported that CD73 regulated suppressive function of Treg cells via activating A2AR which are expressed on target cells. The proliferation of CD4^+^CD25^−^ effect T cells was significantly inhibited by A2AR-specific agonist—CGS21680. And moreover, the expression level of inflammation factors (TNF-α, IL-2, IFN-γ, IL-13, IL-4, IL-1α, IL-1β) was also significantly inhibited by CGS21680 treatment [20]. Ohta et al. [22] found that A2AR agonist CGS21680 promoted Treg cells proliferation, while A2AR antagonist ZM241385 inhibited Treg cells proliferation. These data suggested that the activity of adenosine pathway was necessary for suppressive activation of Treg cells. Moreover, adenosine has been shown to regulate the differentiation of macrophage through A2A and A2B receptors. Adenosine increases alternative-macrophage activation, which includes various anti-inflammatory macrophage phenotypes, by inhibiting M1-macrophage activation and promoting M2-macrophage polarization [23]. Adenosine can also regulate dendritic cell differentiation and function. The stimulation of A2AR can decrease MHC-II expression and deregulate the antigen-presentation activation of dendritic cells, which can furthermore reduce immune response [24]. Taken together, these results demonstrated that adenosine pathway played a key role in the suppression of inflammatory response.

## Adenosine pathway changes in human autoimmune diseases

Development of autoimmune diseases is associated with the deficiency of immune-suppressive signal, in which the adenosine has been demonstrated to play an important role. Therefore, researchers pay attention to study the role of adenosine pathway in autoimmune diseases. Up to date, accumulating data have shown the role of adenosinergic pathway-associated molecules in several types of autoimmune diseases (Table 1).

**Table 1:** Function of adenosine pathway in various autoimmune diseases

AID type	Conclusion	References
Rheumatoid arthritis	Methotrexate unresponsiveness in RA is associated with low expression of CD39 on Treg cells CD73-deficient mice are significantly more susceptible to collagen-induced arthritis, while this increased susceptibility of CD73-deficient mice to CIA is reversed by A2AR activation Serum ADA activity of RA patients is higher compared to that of healthy controls	[26] [25] [27]
System lupus erythematosus	CD39 and CD73 expression levels in Treg cells were decreased in active SLE patients as compared to healthy controls and inactive SLE patients ADA2 activity and total ADA activity are significantly elevated in serum of SLE patients compared to healthy control	[29, 30] [31]
Type I diabetes mellitus	Diminished A1R expression in pancreatic alpha-cells contributes to the pathology of type 1 diabetes A3R activation markedly reduced Beta-TC6 cells proliferation, while this effect was partially blocked by the A3 antagonist TLR9 deficiency promotes CD73 expression in T cells and reduces incidence of TIDM in nonobese diabetic mice	[53] [36] [37]
Multiple sclerosis	CD73-deficient mice are more resistant to experiment autoimmune encephalomyelitis. A2AR antagonist protected WT mice from EAE induction through blockage of A2AR signaling	[38]
Myasthenia gravis	Serum ADA activity of MG patients is significantly higher as compared to normal control Activation of the A2AR attenuates experimental autoimmune myasthenia gravis severity	[54] [45]
Autoimmune hepatitis	In AIH, CD39-positive Tregs are decreased in number and fail to adequately hydrolyze proinflammatory nucleotides and do not efficiently suppress IL-17 production by effector T cells The expressions of CD39 and A2AR were significantly diminished in Th17 cells from autoimmune liver disease patients	[47] [48, 55]
Juvenile idiopathic arthritis	Correlation of low CD73 expression on synovial lymphocytes with reduced adenosine generation and higher disease severity in juvenile idiopathic arthritis	[49]
Autoimmune uveitis	A3R agonist can ameliorate the pathological manifestations of the EAU A2B agonist greatly enhanced the development of EAU	[51] [52]

### Rheumatoid arthritis

Rheumatoid arthritis is a chronic autoimmune disease with joint destruction and severe morbidity. It is characterized by synovial infiltration of inflammatory cells secreting pro-inflammatory cytokines. By using collagen-induced arthritis (CIA) mouse models, which is a commonly used mouse model for RA study, Chrobak et al. found that CD73-deficient mice were significantly more susceptible to CIA than wild-type (WT) mice. CD73 deficiency resulted in an increased production of pro-inflammatory cytokines (IL-1β, IFN-γ, TNF-α, IL-6) in joint and increased joint destruction. In summary, their results demonstrated that CD73 played a protective role in rheumatoid arthritis. In addition, by using A2AR-specific agonist—CGS21680—Chrobak also found that stimulation of A2AR in CD73-deficient mice resulted in arthritis incidence similar to wild-type mice. These data were supported by other independent studies which showed a beneficial effect of CGS21680 on CIA severity in mice models [25].

Methotrexate (MTX) is the standard first-line therapy of rheumatoid arthritis. However, a significant percentage of rheumatoid arthritis patients are resistant to MTX treatment. Peres et al. [26] found that MTX unresponsiveness in rheumatoid arthritis patients was associated with low expression of CD39 on Treg cells and the deceased suppressive activity of Treg cells through reduced adenosine production. These data suggested that low expression of CD39 on Treg cells could be a biomarker for identifying MTX-resistance rheumatoid arthritis patients. Vinapamula et al. [27] have found that serum ADA levels were higher in rheumatoid arthritis patients (*N* = 46) compared to controls (*N* = 46), which suggested that serum ADA could be as inflammatory marker for rheumatoid arthritis patients. However, we recently investigated the serum ADA levels in 60 rheumatoid arthritis patients and 60 healthy controls (age and sex matched, Chinese people), and our results showed that there was no significant difference of serum ADA in RA patients compared to controls (unpublished data). Up to now, ADA is not a biomarker for RA clinical diagnosis. Thus, further studies in larger number of RA patients are needed to evaluate whether serum ADA can be used as a biomarker for RA patients. Taken together, above results supported that adenosinergic pathway was involved in RA development, diagnosis and therapy.

### Systemic lupus erythematosus

Systemic lupus erythematosus is a systemic autoimmune disease with unknown etiology, which affects almost all the organs and tissues. Increasing evidences have suggested that the disturbed T cell homeostasis plays a critical role in the development of SLE. Dolff et al. [28] have reported that imbalance of Treg and Th cells contributes to the pathogenesis of SLE. Valencia found that the Treg cells number and immunosuppressive activation were significantly decreased in the peripheral blood of patients with active SLE as compared to healthy controls and patients with inactive SLE [11]. Li et al. [29, 30] found that the CD39 and CD73 expressions in Treg cells were decreased in active SLE patients as compared to healthy controls and inactive SLE patients. Notably, the adenosine-generatingenzymes expression levels (CD39, CD73) were found to be decreased, while the activity of adenosine degradation enzyme—ADA—was increased in SLE patients. Saghiri et al. [31] showed that total ADA activity and ADA2 activity (one of ADA isoenzymes) were increased in serum of SLE patients and suggested that ADA and its isoenzymes analysis in serum could be used as a useful and noninvasive marker in evaluation of SLE active phase and disease severity. And moreover, the adenosine receptor has been found to be involved in the progression of SLE. By using MRL/lpr mouse model, which shares many characteristics of human SLE, Zhang et al. found that CGS21680 treatment resulted in significant decrease in albuminuria, hematuria and renal immune complex deposition as well as improvement in renal histology. This result demonstrated that A2AR stimulation ameliorated the severity of nephritis and renal vasculitis in MRL/lpr mouse model and, moreover, suggested that A2AR could be considered as a potential therapeutic target for human lupus nephritis [32]. Taken together, above studies demonstrated that adenosine signaling pathway played an important role during SLE progression.

### Type 1 diabetes

Type 1 diabetes mellitus is an organ-specific autoimmune disease caused by immune-mediated destruction of the insulin-producing pancreatic β cells. Accumulating evidences highlight a critical role for the adenosine signaling pathway in the pathophysiology of T1DM [33].

Several studies showed that adenosine could regulate pancreatic β cells homeostasis by controlling its proliferation and regeneration. As we know, pancreatic β cells are biosensors that detect fluctuations of blood glucose levels and release appropriate amounts of insulin when required. In one of these studies, performed in a zebrafish model, Andersson found that adenosine agonist NECA (5′-N-ethylcarboxamidoadenosine) potently increased the regeneration of β cells by promoting their proliferation, and NECA’s effect on β cells proliferation was shown to be mediated by one of zebrafish orthologs of the A2A receptor (called A2aa) [34]. And furthermore, the proliferative and lowering effect of NECA was confirmed in diabetic mice models, suggesting an evolutionarily conserved role for adenosine signaling pathway in β cells proliferation. In addition, another study showed that NECA strongly suppressed expression of the proinflammatory cytokines TNF-α, MIP-1α, IL-12, and IFN-γ in pancreata, splenic cells and T helper 1 lymphocytes, indicating that the benefit of NECA in diabetes therapy was due to immunomodulation [35]. By using mouse pancreatic β-cell line Beta-TC6, Ohtani found that A3 receptor agonist (Cl-IB-MECA) markedly reduced Beta-TC6 cells proliferation, while this effect of A3 agonist was partially blocked by the A3 antagonist [36]. These results suggested that the A3 receptor was involved in modulation of the survival of pancreatic β cells. Moreover, Ohtani also found that adenosine augmented insulin secretion by mouse islets in the presence of either normal or high concentration of glucose via A2A receptor, while this stimulatory effect could be blocked by the treatment with A2A receptor antagonist-SCH58261. Above results suggested that adenosine receptors played an important role in diabetes development and could be potential target for T1DM therapy.

Besides adenosine receptors, CD73, which is a key enzyme for adenosine generation, has also been found to be involved in T1DM development. Tai et al. [37] have showed that TLR9 deficiency in nonobese diabetic mice could significantly protect from T1DM development via up-regulating CD73 expression. This finding indicated that CD73 played an important role in diabetes protection, and might be a therapeutic target for T1DM in humans.

### Multiple sclerosis

Multiple sclerosis is a chronic inflammatory autoimmune disease of the central nervous system (CNS), due to an immune reaction against myelin proteins. The etiology of MS is still unclear. Increasing evidences have suggested that adenosinergic system may be an important factor in MS pathophysiology. Mills et al. demonstrated that CD73-deficient mice were more resistant to experiment autoimmune encephalomyelitis (EAE, which is the classical animal model of MS). In addition, it was observed that the infiltration of lymphocytes into CNS in CD73-deficient mice was fewer than that in WT mice. And moreover, they found that SCH58261 protected WT mice from EAE induction through blockage of A2AR signaling. Lymphocyte infiltration into the CNS was diminished in mice treated with A2AR antagonist or through A2AR knockdown [38]. These findings indicate that the activation of CD73 and A2AR is the promotive factors during EAE development via facilitating lymphocyte migration into CNS. Similarly, A2BR expression is increased in MS patients and EAE mice models, and A2BR-specific antagonists (CVT6883 and MRS-1754) can remit of EAE disease and protect the CNS from immune damage [39]. However, for the other adenosine receptor-A1R, several lines of evidence have showed that A1R may have the protective function in EAE. Firstly, the expression of A1R is reduced in MS patients [40]. Secondly, A1R-deficient mice show more severe forms of progressive and relapsing EAE compared to WT mice [41]. Thirdly, activation of A1R reduces inflammatory response in the CNS [40]. These results demonstrate the different role of adenosinergic pathway-related molecules in MS development.

### Myasthenia gravis

Myasthenia gravis (MG) is a T cell-dependent autoimmune disease characterized by excessive muscle weakness and fatigue. The experimental autoimmune myasthenia gravis (EAMG) rat is a classic animal model of MG, which can be induced by immunization of rats with the acetylcholine receptor (AChR) R97–116 peptide [42]. As early as in 1990, Chiba et al. [43] reported that the serum ADA activity of MG patients (*n* = 30) was significantly higher as compared to that in normal control (*n* = 150). Oliveira’s data showed that serum ADA activity in EMAG animals was significantly higher than that in both control and native animals [44]. We recently investigated the serum ADA activity of MG patients (*n* = 50); however, our results revealed that there was no significant difference between MG patients and healthy controls (unpublished data). Thus, the serum ADA activity should be investigated in more MG patients. Li et al. [45] reported a reduction of A2AR expression in both T cells and B cells residing in spleen and lymph nodes following EAMG induction. A2AR stimulation inhibited anti-AChR antibody production and proliferation of AChR-specific lymphocytes in vitro. Furthermore, preventive treatment of EAMG with CGS21680 was effective in down-modulating disease manifestations and therapeutic treatment partly attenuated the severity of established EAMG. In a recent study, Li et al. [46] evaluated adenosine receptor expression in EAMG rats and found that lymphocyte A1R and A2AR expression levels were decreased, while there was no significant change in A2BR and A3R expressions. Thus, Li et al’s [44] data demonstrated the critical role of A2AR during MG progression and presented the potential opportunities to develop A2AR targeting therapeutic approach. Recently, Oliveira found that CD4^+^CD25^+^FoxP3^+^ regulatory T cells of EAMG expressed lower amount of CD73 as compared to controls. The reduction of CD73 expression and adenosine receptors in EMAG rats strongly suggests that the adenosine signaling pathway may be the dysfunction during MG progression. Thus, stimulation of CD73 or A2AR may have therapeutic potential in MG.

### Autoimmune hepatitis

Autoimmune hepatitis is an inflammatory disease of the liver, characterized by female preponderance, interface hepatitis on histology and autoantibody positivity. Numerical and functional defects of Treg cells are likely to play a permissive pathogenic role in AIH. Several lines of evidence have demonstrated the role of adenosine pathway in AIH. Grant et al. [47] found that the number of CD39^+^ Treg cells was decreased in AIH, which resulted in failure to efficiently suppress IL-17 production by effector T cells. In a recent study, Liberal et al. [48] reported that the expression of CD39 and A2AR was significantly diminished in Th17 cells from autoimmune liver disease patients, and these decreases were associated with impaired generation of immunosuppressive adenosine and defective regulatory properties. These data indicated that adenosinergic pathway involved in AIH development by impairing the immune regulatory function of Treg and Th cells.

### Other autoimmune diseases

Juvenile idiopathic arthritis is a chronic autoimmune rheumatic disease of childhood with unknown etiology, characterized by swelling of the joints and thickening of synovial lining. Botta et al. [49] found that the expression level of CD73 on synovial fluid mononuclear cells (SFMCs) from JIA patients was lower than that in peripheral blood mononuclear cells (PBMCs) from both JIA patients and healthy controls. And moreover, low expression of CD73 on T and B cells in inflamed site is correlated with the clinical severity of JIA patients. This finding suggested that the decreased CD73 expression in SFMCs would lead to a decreased potential for anti-inflammatory activity and then, the deterioration of disease.

Uveitis is an inflammation involving the middle layer of the eye with a high risk of blindness, which can be caused by autoimmune disorder and infection. Experiment autoimmune uveitis (EAU) is a mouse model of endogenous uveitis in humans. In recent years, several studies reported the role of adenosinergic pathway in uveitis by using EAU models. Liang et al. [50] found that γδ T cells expressed different amounts of CD73 during the different stages of EAU. However, the function of CD73 expression in uveitis has not been well demonstrated. Bar-Yehuda et al. found that treatment with the highly selective A3R agonist—CF101—can ameliorate the pathological manifestations of the EAU. This result supported further exploration of CF101 for the uveitis therapy [51]. However, unlike the A3R agonist, Chen et al. [52] showed that selective A2B agonist greatly enhanced the development of EAU, whereas treatment with A2B antagonist significantly ameliorated severity of EAU. Taken together, these data suggested the different effect of the adenosine receptor subtypes involved in uveitis development.

## Conclusion

Autoimmune diseases are caused by disruption of natural immune homeostasis and failure of immune tolerance. Under natural circumstance, the immunosuppressive signal and stimulative signal inter-restrict with each other and maintain the immune homeostasis together. While, the immunosuppressive effects of adenosine present the potential roles of adenosinergic pathway in the development of autoimmune diseases. Indeed, in recent years, increasing data support that adenosinergic pathway plays an important role in various types of autoimmune diseases, and moreover, this discovery shed new light on the diagnosis, monitoring and therapy of certain autoimmune diseases. For diagnosis and monitoring, the increased serum ADA activity in RA and SLE patients present the potential value of serum ADA level that served as a biomarker. For therapy, the connection between low CD39 expression in RA patients and methotrexate unresponsiveness suggests that CD39 expression analysis can be used to identify methotrexate-responsive RA patients. And moreover, some agonist/antagonist of adenosine receptors has displayed favorable curative effect in animal models. Notably, The CF101, which is the specific agonist of A3R, is currently undergoing testing in clinical trials for the treatment of rheumatoid arthritis patients [56, 57]. Although other agonist of adenosine receptors has not been translated into clinical patients, the results in animal models have revealed that adenosine receptors target therapy may be an alternative and realistic therapeutic approach for autoimmune diseases in the future. Thus, future studies aiming at translating adenosinergic pathway target therapy into clinical patients can be expected. Notably, the cardiovascular side effects of a A2AR agonist—GW328267X—have been found in clinical trials of chronic obstructive pulmonary disease treatment [3]. Thus, because of multiple effects of adenosinergic signaling on immune system, although adverse events has not yet been observed in animal models of autoimmune diseases, the potential toxic risk of adenosinergic pathway target therapy cannot be ignored and must be argued carefully before translating into autoimmune disease patients. Up to now the total studies focused on the role of adenosinergic pathway in human autoimmune diseases are still few, and more studies are required to completely evaluate the adenosinergic pathway and its contribution in autoimmune disease development.
